# Benchmarking of Scale-X Bioreactor System in Lentiviral and Adenoviral Vector Production

**DOI:** 10.1089/hum.2019.247

**Published:** 2020-03-17

**Authors:** Hanna M. Leinonen, Saana Lepola, Eevi M. Lipponen, Tommi Heikura, Tiina Koponen, Nigel Parker, Seppo Ylä-Herttuala, Hanna P. Lesch

**Affiliations:** ^1^ Kuopio Center for Gene and Cell Therapy, Kuopio, Finland; ^2^ FinVector, Kuopio, Finland; and; ^3^ Molecular Medicine, A.I. Virtanen Institute for Molecular Sciences, University of Eastern Finland, Kuopio, Finland.

**Keywords:** virus production, bioreactor, fixed-bed, iCELLis bioreactor, lentivirus, scale-X bioreactor

## Abstract

We have previously produced viral vectors (lentiviral vector, adenoviral vector, and adeno-associated viral vector) in small and in commercial scale in adherent cells using Pall fixed-bed iCELLis^®^ bioreactor. Recently, a company called Univercells has launched a new fixed-bed bioreactor with the same cell growth surface matrix material, but with different fixed-bed structure than is used in iCELLis bioreactor. We sought to compare the new scale-X™ hydro bioreactor (2.4 m^2^) and iCELLis Nano system (2.67 m^2^) to see if the difference has any effect on cell growth or lentiviral vector and adenoviral vector productivity. Runs were performed using parameters optimized for viral vector production in iCELLis Nano bioreactor. Cell growth was monitored by counting nuclei, as well as by following glucose consumption and lactate production. In both bioreactor systems, cells grew well, and the cell distribution was found quite homogeneous in scale-X bioreactor. Univercells scale-X bioreactor was proven to be at least equally efficient or even improved in both lentiviral vector and adenoviral vector production. Based on the results, the same protocol and parameters used in viral vector production in iCELLis bioreactor can also be successfully used for the production in scale-X bioreactor system.

## Introduction

Viral vectors for gene therapy are still mainly produced using adherent cells. Standard small-scale manufacturing has relied on different flask approaches, and scale-up options have been, for example, Cell Factories (Thermo Fisher Scientific) or Hyperstacks (Corning). However, they require a lot of manual handling, may need open connections, and are not monitored or controlled for pH, dissolved oxygen, and so on.^1^ Thus, there has been a need for large-scale, disposable bioreactor for adherent cells. ATMI/Pall brought iCELLis^®^ fixed-bed technology on market about a decade ago. The three-dimensional fixed-bed of iCELLis Nano bioreactor consists of hundreds of small 13.9 cm^2^ size polyethylene terephthalate (PET) fibers (“carriers”) packed inside the bioreactor. iCELLis bioreactors are available both in high (144 g/L) and low (96 g/L) compaction. The culture area of iCELLis Nano bioreactor is up to 4 m^2^, which is a valuable tool for small-scale batches, but can mainly be used for process development and optimization. iCELLis 500 is the commercial scale system with culture area from 66 to 500 m^2^, depending on the fixed-bed height and carrier compaction.

Our team was one of the first who implemented iCELLis technology for HEK293(T)-adherent cell-based manufacturing process for adenoviral,^2^ lentiviral,^3,4^ and adeno-associated viral (AAV)^5^ vectors. Process development was performed in iCELLis Nano bioreactor, processes were scaled up to iCELLis 500 scale, and currently we have been producing viral vector material in iCELLis 500 for clinical trials.^6^ We have been able to produce more than 1 × 10^16^ adenoviral particles per batch. Others have also found iCELLis bioreactor useful for retrovirus,^7^ AAV,^8^ Rabies,^9^ Hepatitis-A,^9^ and Chikungunya^9^ vaccines, or for recombinant protein production in insect cells.^10^ iCELLis 500 bioreactor is good manufacturing practice (GMP) compliant, fully disposable, and controlled system with perfusion capability. It supports adherent cell growth and high titer production.

Given our expertise with iCELLis system, we were naturally interested in investigating a recent adherent bioreactor offering by Univercells. Univercells' scale-X™ bioreactor system is an automated, single-use fixed-bed bioreactor, which has culture area from 2.4 m^2^ (commercial name known as hydro) to 600 m^2^ (nitro) and above. Univercells also provides 10–30 m^2^ “mid-size” scale-X carbo bioreactor systems. All scale-X bioreactors should be available for GMP manufacturing next year. They are suitable, for example, for *ex vivo* use when the yield requirements are lower than in direct viral vector administration into the patient. The fixed-bed material is the same, but bed structure is different compared to iCELLis fixed-bed. While the iCELLis fixed-bed is relatively randomly packed with macrocarriers, in the scale-X bioreactor fixed-bed is a consistent form of nonwoven spiral-wound double-layer PET with a spacer netting between the layers. Such bed structure might allow better, more homogenous cell distribution throughout the fixed-bed. In addition to viral vector production, scale-X bioreactor system enables also continuous in-line concentration due to hollow fiber tangential-flow filtration option built in the system. Moreover, by combining scale-X bioreactors with the NevoLine™ microfacilities, that is, chained closed cabinets for bioreactors and in-line downstream processing, the GMP facility requirements could be lower. We tested the new scale-X hydro bioreactor system for lentiviral and adenoviral vector manufacturing, to determine if the different membrane matrix assembly has effect on cell growth or viral vector productivity, and compared the system to the iCELLis bioreactor. The same parameters that were previously optimized for iCELLis bioreactor were used for both bioreactor systems.^2,4^ Cell growth was found similar in both bioreactors. Productivity in scale-X hydro bioreactor was proven to be at least equally efficient as in iCELLis Nano system.

## Materials and Methods

### Cell lines and culturing media

293T (ATCC, Manassas, VA) and HEK293 (ATCC) cells cultivated in high- or low-glucose Dulbecco's modified Eagle's medium (DMEM; Gibco, Paisley, United Kingdom/Sigma-Aldrich, Irvine, United Kingdom) supplemented with 10% (v/v) fetal bovine serum (FBS; Gibco) and 50–100 U/mL penicillin, 50–100 μg/mL streptomycin (Gibco), and 4 mM l-glutamine (Gibco) were used for both lentiviral vector and adenoviral vector production. In addition, in lentiviral vector production, post-transfection (PT) media were supplemented also with 1 × nonessential amino acids (Gibco), 1 mM sodium pyruvate (Gibco), and 1:500 CD-lipid supplement (Gibco). FBS was included in culturing media starting during cell expansion before bioreactor inoculation, and in bioreactor runs until 24 h PT, after which runs continued without FBS. Seven thousand to 9,000 cells/cm^2^ were inoculated. Before inoculation, all cells were cultivated in T-flasks in humidified environment at +37°C and 5% CO_2_.

HeLa cells (ATCC) required for infective titer analysis of lentiviral vector were cultured in DMEM—10% FBS (Gibco)—50 U/mL penicillin and 50 μg/mL streptomycin (Gibco). FBS was not included during transductions.

### Lentiviral vector production in iCELLis Nano and scale-X hydro bioreactors

Altogether, four scale-X bioreactor runs were performed. In three runs, lentiviral vectors were produced, whereas in one of the runs, bioreactor was dismantled according to instructions provided by Univercells before transfection to analyze cell densities in different areas of the fixed-bed. One iCELLis Nano bioreactor was run parallel as a control.

**Figure 1.**
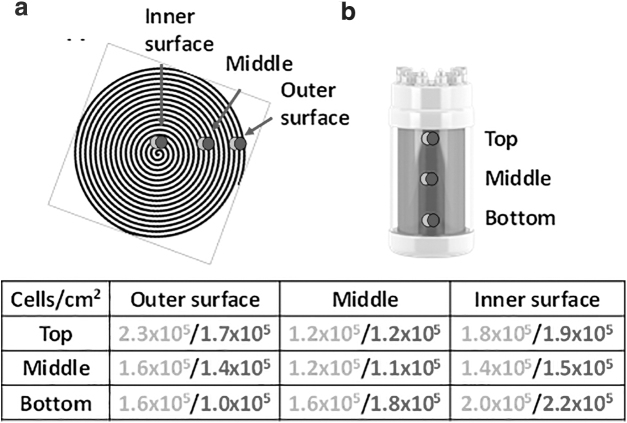
Cell counts of dismantled Univercells' scale-X™ hydro bioreactor. Schematic picture of scale-X bioreactor from *top* **(a)** and from the *side* **(b)**. *Light gray* and *dark gray* spots indicate the points from which samples were taken for cell density analysis. *Light gray* = sample of inner membrane, and *dark gray* = sample of outer membrane layer. For cell density analysis, nuclei were counted from *top*, *middle* and, *bottom* of the fixed-bed, and from the outer surface, *middle*, and inner surface of the rolled membrane. Cell densities (cells/cm^2^) are shown in the table below the schematic pictures.

Lentiviral vector production in iCELLis Nano was performed using a 2.67 m^2^ low compaction fixed-bed (Pall Life Sciences, Hoegaarden, Belgium) bioreactor. In scale-X bioreactor runs, Univercells' (Gosselies, Belgium) 2.4 m^2^ scale-X hydro bioreactors were used. Runs were performed as previously described,^4^ targeting 0.5 g/L glucose by perfusion. Glucose and lactate were measured once or twice a day (Cedex-Bio; Roche, Mannheim, Germany). Nonattached cells were counted from a bioreactor media sample 1 h after inoculation. Nuclei of cells attached to carriers in iCELLis Nano bioreactor were counted on days 1–4 as previously reported.^2,3^ In scale-X hydro bioreactor, there are sampling strips (approximately the same size as carriers in iCELLis Nano bioreactor) located between membrane layers that can be sampled and nuclei counted similar to iCELLis Nano system.^2,3^ For each nuclei count, two strips were picked using sterile tweezers. In addition, one scale-X bioreactor fixed-bed was dismantled, and nuclei were counted from top (1 cm from the top edge), middle, and bottom of the bed (1 cm from the bottom), from both membrane layers, and from the outer, middle, and inner surface of the fixed-bed (Fig. 1). For those nuclei counts, 1 cm^2^ pieces were cut from the membrane.

In all runs, third-generation LV-GFP^3^ was produced using PEIpro^®^ (Polyplus-transfection, Illkirch, France)-mediated transfection with DNA:PEI ratio of 1:1 and 200 ng/cm^2^ of plasmids (PlasmidFactory Bielefeld, Germany). Transfection was performed as described before.^3,4^

A complete medium change was performed before starting harvest, and viral vector was harvested by collecting perfused media in RT between 24 and 72 h PT. In the end of the run, bioreactors were drained into the corresponding collection bags.

### Adenoviral vector production in iCELLis Nano and scale-X hydro bioreactors

Altogether, two bioreactor runs were performed, one with scale-X bioreactor and one with iCELLis Nano system, to compare differences in adenoviral vector productivity. Runs were performed as previously described,^2^ except targeting 0.5 g/L glucose by perfusion as in lenti runs.^4^ Cells were inoculated using a density of 7,000 cells/cm^2^. Similar to lenti runs, glucose and lactate were measured twice a day (Cedex-Bio), and nuclei were counted on days 1–4. Infection was performed as described before^2^ using the same amount of adenoviral vector (Ad-GFP^11^) in both bioreactors (with average multiplicity of infection value of 75). Cell lysis was performed 68 h after infection with detergent-based lysis.^2^ Harvest material was clarified using 0.027 m^2^ DEPTH filters (Millipore, Billerica, MA).

### Analytics

Infective titers of lentiviral vectors (transducing units [TU]/mL) were determined using a quantitative polymerase chain reaction (qPCR)-based method.^4^ Lentiviral vector particle (vp) titer was analyzed by converting pg/mL results of p24 enzyme-linked immunosorbent assay (ELISA; PerkinElmer, Waltham, MA) to vp/mL by assuming 12,500 lentiviral particles per 1 pg of p24.^12,13^ Adenoviral vector particle titer was analyzed with high-performance liquid chromatography (HPLC).^2,14^

## Results and Discussion

The first fully integrated, disposable fixed-bed bioreactor, iCELLis, was launched by ATMI/Pall ∼10 years ago. In addition, some other adherent bioreactors have been developed, such as Celligen (NewBrunswick Scientific),^15^ CellCube (Costar),^15^ packed-bed bioreactors (BioBLU; Eppendorf),^16^ and microcarrier-based bioreactors.^17^ Among the latest inventions is scale-X bioreactor manufactured by Univercells, a company that is co-founded by José Castillo, also known as the developer of iCELLis system.^18^ Our team has plenty of experience on viral vector production in iCELLis bioreactor.^2–5^ Because the membrane to which cells attach in iCELLis and scale-X bioreactors is the same material, we hypothesized that parameters optimized and used in iCELLis bioreactor would be rather easy to transfer to scale-X bioreactor.^4^ For testing the hypothesis, we compared the cell growth, cell distribution, and medium consumption, as well as lentiviral and adenoviral vector production in scale-X bioreactor to iCELLis Nano system.

### Cell distribution

We have previously compared cell distribution in high compaction fixed-bed of iCELLis Nano bioreactor (4 m^2^) to low compaction fixed-bed (2.67 m^2^), and found cells more equally distributed in low compaction.^3^ In high compaction fixed-bed, there were large differences in cell densities depending on from which layer cells were counted (threefold to fourfold more cells in the bottom compared to top). Although variability was smaller in low compaction bed, we noted twofold to threefold more cells in the middle of the low compaction bed compared to the bottom. Cell density analysis was made for dismantled scale-X bioreactor to see how the cells are located in different parts of the fixed-bed (Fig. 1). Cells were found relatively equally distributed throughout the fixed-bed (Fig. 1). However, cell density was approximately twofold higher in the middle of the fixed-bed both when analyzed vertically or horizontally, which is close to what has been observed in low compaction iCELLis bioreactor. Differences were minor when cell densities of outer and inner membrane of the double-layered membrane were analyzed. It is likely that the rolled membrane-like structure of scale-X bioreactor fixed-bed causes less variation in cell densities and cell growth between batches. iCELLis bioreactors have larger variation in cell densities, especially in high compaction, compared to scale-X bioreactor, likely due to relatively random and tight packing of the carriers. Therefore, in iCELLis bioreactor, there is always variability between each bioreactor with some less dense and some more dense areas.

Moreover, in sampling strips of the dismantled bioreactor taken just before dismantling, ∼1.1 × 10^5^ cells/cm^2^ were calculated, which is close to the densities found in top-middle (1.2 × 10^5^ ± 0.4 × 10^4^) of the membrane (Fig. 1), in the position where the carriers were located. Therefore, it seems that the sampling strips in scale-X bioreactor are representative, and can be used for evaluating the cell density.

### Cell growth

For iCELLis Nano system, we have optimized the cell density used in inoculation,^3^ and thus, 7,000 293T cells/cm^2^ were inoculated in control Nano on day 0. For scale-X bioreactors two different inoculation densities, 7,000 cells/cm^2^ (scale-X bioreactor runs 1–3) and 9,000 cells/cm^2^ (scale-X bioreactor run 4), were used. Cells were found to attach to the PET membrane as fast in both bioreactor types, because 1 h post-inoculation, no free cells were found from media sample taken from the bioreactors. Target cell density during transfection was 150,000–200,000 cells/cm^2^ and typically, in iCELLis Nano runs, that density is reached in 4 days.^3^ For monitoring cell growth, both iCELLis Nano and scale-X bioreactors can be opened inside laminar flow hood, and carriers (Nano bioreactor) or sampling strips (scale-X bioreactor) can be picked from the bioreactors using sterile tweezers. Importantly, there is also a sampling possibility of strips available in the larger fixed-bed sizes of scale-X bioreactor system, while carriers from iCELLis 500 bioreactor cannot be sampled.

**Figure 2.**
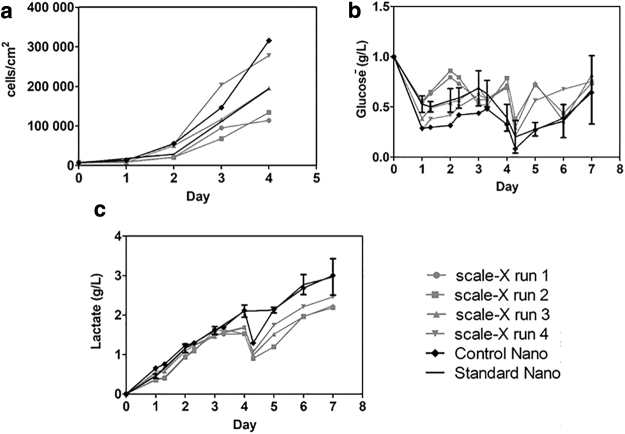
Cell density and glucose and lactate concentrations in scale-X hydro bioreactor and iCELLis^®^ Nano bioreactor runs. **(a)** Cell densities (cells/cm^2^) calculated from *top* of the fixed-bed on days 0–4, **(b)** glucose and **(c)** lactate concentrations on days 0–7. Control Nano = Nano run, run together with scale-X bioreactor runs. Standard Nano = average of five standard Nano runs. scale-X 1–4 = scale-X bioreactor runs 1–4.

Nuclei of carriers/sampling strips sampled from the top of the fixed-bed of bioreactors on days 1–4 (before transfection) were calculated and were converted to cell densities (Fig. 2a). Targeted cell density was reached in scale-X bioreactor run 3, and also in scale-X bioreactor runs 1 and 2, cell density was almost on target. Higher inoculation cell density in scale-X run 4 resulted in too high cell density during transfection. Likely due to uneven cell distribution throughout the fixed-bed, targeted cell density was exceeded also in the control Nano run.

### Glucose and media consumption

**Figure 3.**
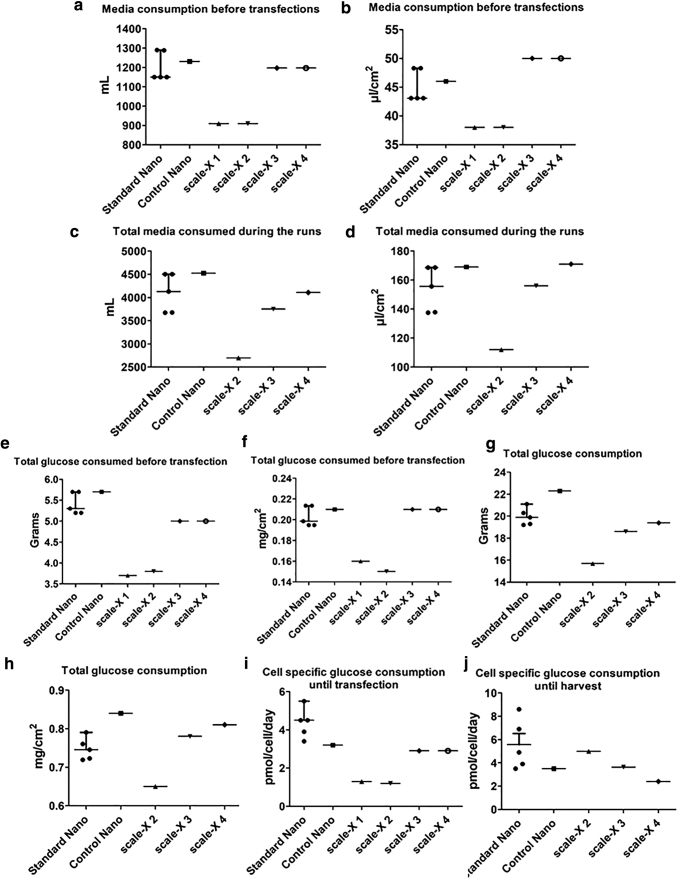
Scatter dot blot images of media and glucose consumption in scale-X hydro bioreactor and iCELLis Nano bioreactor runs. **(a, b)** Media consumption before transfections in mL **(a)** and in μL/cm^2^ **(b)**. **(c, d)** Total media consumed during the runs until harvest in mL **(c)** and in μL/cm^2^ **(d)**. **(e**, **f)** Glucose consumed until transfection in g **(e)** and in mg/cm^2^ **(f)**. **(g, h)** Total glucose consumption until harvest in g **(g)** and in mg/cm^2^ **(h)**. **(i, j)** Cell-specific glucose consumption in pmol/cell/day until transfection **(i)** and until harvest **(j),** taking into account that after transfection, cell density is not increased. Standard Nano = five standard iCELLis Nano runs aiming at lentiviral vector production, control Nano = run together with scale-X bioreactor runs. scale-X 1–4 = scale-X bioreactor runs 1–4, of which run 1 was not transfected, but run was stopped on day 4 to disassemble the fixed-bed. For standard Nano runs value for each runs is indicated as a separate spot; in addition, SEM and median are shown. SEM, standard error of the mean.

In all runs, perfusion was applied to supply fresh media. Aim was to maintain 0.5 g/L of glucose concentration in bioreactor by using high-glucose DMEM as perfusion media.^3,4^ For adjusting perfusion rate, both glucose and lactate concentrations were measured daily from the bioreactor media (Fig. 2b, c). Although in scale-X bioreactor runs 1 and 2, both media and glucose consumptions were lower compared to standard iCELLis Nano runs, in runs 3 and 4, glucose and media consumption were in an iCELLis Nano range (Fig. 3). It needs to be taken into account that in run 4, more cells/cm^2^ were used in inoculation than in other runs. Cell-specific glucose consumption was up to fourfold lower in all scale-X runs compared to control or standard iCELLis Nano runs when calculated before transfection (Fig. 3i). Taking into account that cell density is not changed after transfection, estimations of cell-specific glucose consumption were calculated also until harvest (Fig. 3j). Cell-specific glucose consumption was found up to threefold lower in scale-X bioreactor runs compared to standard iCELLis Nano runs. However, it must be taken into account that cell densities vary throughout the fixed-bed in both bioreactor types, and cell-specific glucose consumptions are only calculated based on sampled top carriers. This reflects to lower media/glucose consumption in scale-X bioreactors. Especially if the perfusion rate before 24 h PT is reduced, then expenses are lowered even more because smaller volume of expensive FBS is required (in our protocol, FBS is not used in perfusion media starting 24 h PT). In addition, smaller media consumption in perfusion decreases the harvest volume, which is beneficial for downstream processes. Reason for the smaller glucose/media consumption in scale-X bioreactor can only be speculated, but it may partly be due to the more homogeneous fixed-bed in scale-X. Moreover, in scale-X bioreactor, cells might be more equally reached by the circulating media.

Based on total media consumption in iCELLis Nano and scale-X, if directly scaled up to iCELLis 500 with 333 m^2^ fixed-bed, a total of ∼510 L media would be used in perfusion, and the volumes in scale-X nitro with 600 m^2^ fixed-bed would be 670–940 L.

### Lentiviral vector yields in iCELLis Nano and scale-X bioreactors

**Figure 4.**
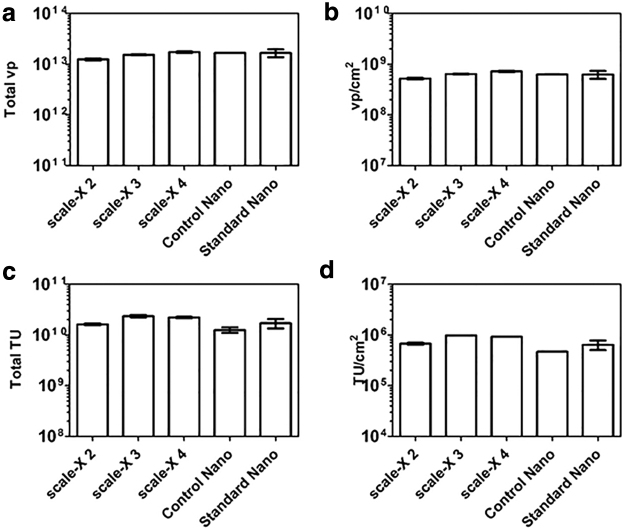
Lentiviral vector yields in scale-X hydro bioreactor and iCELLis Nano bioreactor runs. **(a, b)** Total lentiviral particles (vp) and vp/cm^2^ produced, respectively, in scale-X bioreactor and iCELLis Nano bioreactor runs. **(c, d)** Total TU and TU/cm^2^ produced, respectively, in scale-X bioreactor and iCELLis Nano bioreactor runs. Control Nano = Nano run, run together with scale-X bioreactor runs. Standard Nano = average of five standard Nano runs. scale-X 1–4 = scale-X bioreactor runs 1–4. Mean ± SEM. TU, transducing units; vp, vector particle.

In the scale-X bioreactor and control/standard Nano runs, both viral particle titer analyzed by p24 ELISA and infective titer analyzed by qPCR-based method^4^ were in the same range (Fig. 4a–d). Infective titers analyzed in separate assays cannot reliably be compared to each other,^4,19^ but when titered at the same time, comparisons can be made. Control Nano and scale-X bioreactor runs 3 and 4 were titered simultaneously, and in those runs, almost two times more TU were produced in scale-X bioreactor runs compared to iCELLis Nano run. Because the fixed-bed size in scale-X bioreactor is smaller compared to iCELLis Nano, the TU difference per cm^2^ was even larger (Fig. 4d). Vp yields were close to each other in all runs (Fig. 4a, b), and thus, based on these runs, vp/TU ratio in scale-X bioreactor run 3 (vp/TU: 652) seemed to be better compared to iCELLis Nano (vp/TU: 1,339) bioreactor. However, during transfection, according to nuclei count, cell density in iCELLis Nano bioreactor was approximately double than targeted. As the same amount of cells were inoculated as in standard runs, this might be explained by uneven distribution of cells in fixed-bed, which may have reduced transfection efficiency and therefore also productivity. As seen in scale-X bioreactor run 4, to which 9,000 cells/cm^2^ were inoculated, larger cell density, at least, does not increase productivity, and 7,000 cells/cm^2^ seems to be optimal for inoculation also in scale-X bioreactor. Because productivity in all scale-X bioreactor runs was, at least, similar or even higher compared to iCELLis Nano (both control and standard runs), it seems that it is relatively easy to transfer parameters used in iCELLis Nano bioreactor to scale-X bioreactor, without a need to optimize the parameters again for another kind of fixed-bed. However, optimization may further increase the titers.

We have noted earlier that lentiviral vector yield/cm^2^ is lower when produced in high compaction fixed-bed compared to low compaction bed.^3^ The same amount of viral vector was found to be produced in iCELLis Nano bioreactor in 2.67 m^2^ low compaction fixed-bed as in 4 m^2^ high compaction fixed-bed. Moreover, the media consumption was lower and was easier to predict when low compaction bed was used.^3^ Thus, with the current iCELLis 500, theoretically up to a total of 1–3 × 10^12^ TU (when titered in our HeLa cells using qPCR-based method^4^) or a total of 1–3 × 10^15^ vp (viral particles) can be produced. At the moment, the largest low compaction iCELLis bed size is 333 m^2^. From scale-X bioreactor, only one compaction is available. Based on manufacturer of scale-X bioreactor, the largest bed sizes available are at least 600 m^2^. Thus, if directly scaled up, by using scale-X bioreactor system, the amount of viral vector produced in iCELLis bioreactor could be, at least, doubled or even tripled. However, we have shown that, although iCELLis bioreactor system should be directly scalable, for unknown reason, when lentiviral vector production is scaled up from iCELLis Nano scale to iCELLis 500 scale (using 100 or 333 m^2^), even more viral vector/cm^2^ is produced compared to smaller scale, and interestingly, media consumption in large scale/cm^2^ is smaller.^4^ Therefore, if productivity improvement in large scale is not related to the composition of the bioreactor bed, but rather to other large-scale process parameters such as antibiotic-free production, and the same occurs also in large-scale scale-X bioreactor, it is possible that >1 × 10^13^ TU (titered in our HeLa cells) or >1 × 10^16^ vp are achieved in scale-X nitro bioreactor.^1^

As both iCELLis and scale-X bioreactors are intended for adherent use, it is true that they have limited scalability. With suspension bioreactor scalability is less limited, and, for example, the use of 2,000 L suspension bioreactor would massively increase productivity. Still, many lentiviral vector batches, even for clinical trials, are manufactured using cell factories,^20,21^ 3-L stirred tanks,^22–24^ or only up to 50-L wave bioreactor or stirred tanks.^25,26^ However, only few viral vector producers have reported the use of larger suspension bioreactors.^27^ In adherent bioreactors such as iCELLis and scale-X bioreactor systems, fresh media are easily provided and used media are removed by perfusion. Because lentiviral vector is produced into media, perfusion makes it easy to collect the viral vector, and enables continuous downstream processing. Although there are perfusion options for suspension bioreactor, perfusion in suspension is still early in its development and appropriate perfusion devices for lentiviral vector production still require optimization or demonstration at clinical/commercial scale.^28,29^ In addition, foam formation in suspension bioreactors can be problematic for viral vector production.^30,31^ Also, cells grown in serum-free media or even cells adapted to suspension cultures could be grown in fixed-bed bioreactors as cells nevertheless will be entrapped to the membrane structures of the fixed-bed. Thus, the advantage of easy and gentle perfusion without any additional device could be exploited also in serum-free conditions, and suspension-adapted cells by using fixed-bed bioreactor.

Already, when scaled up to scale-X nitro bioreactor (600 m^2^), depending on the perfusion rate, 400–600 L of lentiviral vector with >10^6^ TU/mL (when titered in our HeLa cell line^4^) and >10^9^ vp/mL could theoretically be produced using our current parameters. That is comparable to 200-L stirred tank bioreactor with fed-batch in which 0.5–5 × 10^7^ TU/mL infective titers have been achieved.^25,32^ However, without a lentiviral vector reference standard, the infective titers cannot fully be compared between laboratories/production sites. Moreover, often, lentiviral vector productivity in adherent cells is higher compared to suspension production. This can be due to different cell culturing media because certain media support transfection and productivity better than others.^33^ This is also partly due to the presence of FBS, which is still commonly used in adherent production (in our protocol, until 24 h PT^3,4^). In addition, not all the cell lines that produce lentiviral vector grow well in suspension. Large lentiviral vector harvest volumes may be difficult downstream processed, or would preferably require continuous downstream processing. Every lentiviral vector producer should carefully think what volume of vector is required and how easily and fast the fragile lentiviral vector can be downstream processed. Already, with the amount of viral vector that could be produced in scale-X nitro (600 m^2^) bioreactor, even after only 10% recovery after downstream processing, from hundreds^34^ up to thousands^35,36^ of doses could be obtained, depending on the application. However, for sure, for the production of other viral vectors, such as AAV, larger bioreactors are needed, as the required viral vector numbers per patient are often higher compared to lentiviral vectors.^8,37,38^

### Adenoviral vector production in iCELLis Nano and scale-X bioreactors

In addition to lentiviral vectors (Fig. 4), we also compared the production of adenoviral vectors in iCELLis bioreactor compared with scale-X bioreactor. Titers for AdGFP^11^ produced in scale-X hydro system and iCELLis Nano bioreactors were analyzed using HPLC.^2^ The titer for scale-X bioreactor run was 1.11 × 10^11^ viral particles per milliliter and for the iCELLis Nano run 8.53 × 10^10^ viral particles per milliliter after clarification. Results indicate that scale-X bioreactors can also be used to produce adenoviral vectors with equally good yield compared to iCELLis system.

## Conclusion

Univercells' scale-X fixed-bed bioreactor has proven to be efficient for viral vector manufacturing. Cell growth was monitored by nuclei count, glucose consumption, and lactate production. Feeding strategy was based on perfusion. In scale-X bioreactor, cells were growing well and the cell distribution was relatively homogenous through the spiral-wound fixed-bed. Lentiviral vector productivity in scale-X hydro system was efficient, and similar or even higher yields (a total of 2.4 × 10^10^ TU, *i.e.*, 9.8 × 10^5^ TU/cm^2^) of lentiviral vectors were produced in scale-X bioreactor compared to iCELLis Nano. In control iCELLis Nano, 1.3 × 10^10^ TU (4.7 × 10^5^ TU/cm^2^), and in standard iCELLis Nano, 1.7 × 10^10^ ± SD 8.7 × 10^9^ TU ( = 6.4 × 10^10^ ± SD 3.4 × 10^5^ TU/cm^2^) were obtained. Also, adenoviral vector productivities were similar between iCELLis Nano (8.53 × 10^10^ vp/mL) and scale-X hydro (1.11 × 10^11^ vp/mL) bioreactors. Thus, it seems that parameters for viral vector production from iCELLis bioreactor can be easily transferred into scale-X bioreactors, and vice versa.
